# 594. An Open-Label Study in Healthy Volunteers to Determine the Absolute Bioavailability of, the Effect of Food and Dosing by Nasogastric Tube upon the Pharmacokinetics of a Single Oral Dose of Olorofim (OLO)

**DOI:** 10.1093/ofid/ofac492.646

**Published:** 2022-12-15

**Authors:** Karen Cornelissen, Paul A Newell, John H Rex, Hal Galbraith, Samuel Israel, James Bush, M B ChB

**Affiliations:** F2G, Manchester, England, United Kingdom; F2G, Manchester, England, United Kingdom; F2G, Limited, WELLESLEY HILLS, MA; IQVIA, Lees Summit, Missouri; LabCorp CRU, leeds, England, United Kingdom; Labcorp, Leeds, England, United Kingdom

## Abstract

**Background:**

OLO is a novel antifungal active vs. *Aspergillus* (including azole-resistant strains), resistant moulds (e.g., *Lomentospora prolificans*), and dimorphic moulds. Information on absolute bioavailability and effect of food upon the pharmacokinetics (PK) of OLO when administered as a 30 mg tablet, including feasibility of alternative enteral delivery, is required for appropriate guidance on dosing.

**Methods:**

Healthy male and female subjects in Cohorts A and B (6/cohort; high carbohydrate /low fat meals at 8 and 24 h for Cohort A and low carbohydrate/high fat meals for Cohort B ) participated in 3 treatment periods; single doses of 150 mg OLO as a 2-h IV infusion, a fasted PO dose (after overnight fast), and a fed single PO dose (30 min after starting a high fat breakfast). Healthy subjects in Cohort C participated in 2 treatment periods and received fasted single doses of 150 mg OLO either PO as intact tablets or via NG tube (tablets dispersed in water). PK sampling was performed for 120 h after each dose, with at least 10 days between dosing occasions per subject.

**Results:**

The absolute bioavailability of a fasted single PO dose of OLO was found to be 67%. Administering OLO in the presence of food resulted in slight reduction in overall exposure, with an approximate 30% reduction in C_max_ (Table 1). Multiple post-dose OLO peaks were observed for most subjects regardless of dose route or fed state; peaks typically coincided with meal times (Figure).

OLO systemic exposure was similar for intact tablets given PO and tablets dispersed in water given via NG tube (Table).

**Statistical Analysis of Olorofim PK parameters for Cohorts A, B and C**

**Conclusion:**

OLO (30 mg tablet) has good oral bioavailability and based upon the minimal impact of a high-fat breakfast upon overall exposure, can be administered with or without food. No dose adjustment is needed if switching from oral dosing with intact tablets to dosing tablet in water via NG tube.

**Disclosures:**

**Karen Cornelissen, PhD**, F2G: Employee **Paul A. Newell, MB BS**, AstraZeneca: Stocks/Bonds|F2G: Stocks/Bonds **John H. Rex, MD**, Advent Life Sciences: Operating Partner|Advent Life Sciences: Ownership Interest|AMR Action Fund: Advisor/Consultant|AstraZeneca: Stocks/Bonds|Basilea Pharmaceutica: Advisor/Consultant|Bugworks Research, Inc.: Advisor/Consultant|F2G, Limited: Employee|F2G, Limited: Stocks/Bonds|Forge Therapeutics: Advisor/Consultant|GlaxoSmithKline: Advisor/Consultant|Pfizer Pharmaceuticals: Honoraria|Sumitovant: Advisor/Consultant **Samuel Israel, MBBS**, Labcorp: Eemployee **James Bush, MB ChB, PhD**, Labcorp: Employee (Salaried)|Labcorp: Stocks/Bonds.

